# Combination of Bioactive Polymeric Membranes and Stem Cells for Periodontal Regeneration: *In Vitro* and *In Vivo* Analyses

**DOI:** 10.1371/journal.pone.0152412

**Published:** 2016-03-31

**Authors:** Flávia Gonçalves, Míriam Santos de Moraes, Lorraine Braga Ferreira, Ana Cláudia Oliveira Carreira, Patrícia Mayumi Kossugue, Letícia Cristina Cidreira Boaro, Ricardo Bentini, Célia Regina da Silva Garcia, Mari Cleide Sogayar, Victor Elias Arana-Chavez, Luiz Henrique Catalani

**Affiliations:** 1 Departamento de Química Fundamental, Instituto de Química, Universidade de São Paulo, São Paulo, SP, Brasil, 05508–000; 2 Departamento de Fisiologia, Instituto de Biociências, Universidade de São Paulo, São Paulo, SP, Brasil, 05508–090; 3 Departamento de Biomateriais e Biologia Oral, Faculdade de Odontologia, Universidade de São Paulo, São Paulo, SP, Brasil, 05508–000; 4 NUCEL/NETCEM—Núcleo de Terapia Celular e Molecular, Faculdade de Medicina, Universidade de São Paulo, São Paulo, SP, Brasil, 05360–130; 5 Departamento de Bioquímica, Instituto de Química, Universidade de São Paulo, São Paulo, SP, Brasil, 05508–000; Boston University, UNITED STATES

## Abstract

Regeneration of periodontal tissues requires a concerted effort to obtain consistent and predictable results *in vivo*. The aim of the present study was to test a new family of bioactive polymeric membranes in combination with stem cell therapy for periodontal regeneration. In particular, the novel polyester poly(isosorbide succinate-co-L-lactide) (PisPLLA) was compared with poly(L-lactide) (PLLA). Both polymers were combined with collagen (COL), hydroxyapatite (HA) and the growth factor bone morphogenetic protein-7 (BMP7), and their osteoinductive capacity was evaluated via *in vitro* and *in vivo* experiments. Membranes composed of PLLA/COL/HA or PisPLLA/COL/HA were able to promote periodontal regeneration and new bone formation in fenestration defects in rat jaws. According to quantitative real-time polymerase chain reaction (qRT-PCR) and Alizarin Red assays, better osteoconductive capacity and increased extracellular mineralization were observed for PLLA/COL/HA, whereas better osteoinductive properties were associated with PisPLLA/COL/HA. We concluded that membranes composed of either PisPLLA/COL/HA or PLLA/COL/HA present promising results *in vitro* as well as *in vivo* and that these materials could be potentially applied in periodontal regeneration.

## Introduction

Periodontal disease is characterized by an inflammatory reaction and loss of dental support tissues [1]. Therefore, periodontal treatments primarily aim to eliminate the inflammatory process and to reestablish periodontal regeneration. In the early 1980s, the concept of guided tissue regeneration (GTR) was developed, which involves the use of an occlusive membrane to prevent the growth of epithelial tissue inside a periodontal defect, allowing the cells of the periodontal ligament to regenerate at the site [2]. The membrane was prepared from polytetrafluoroethylene, a biocompatible but non-degradable polymer. However, bacterial contamination and the need for a second surgery to remove the membrane were two of the primary concerns associated with this technique [3,4]. Currently, absorbable membranes made of collagen (COL) or biodegradable polyesters such as poly(L-lactide) (PLLA) and poly(lactic-co-glycolic acid) (PLGA) are commercially available [2]. Although PLLA and PLGA have slower degradation [3] and better mechanical properties than COL membranes [5,6], these materials have low cell affinity [7]. COL membranes, which show higher cell affinity, are thus the most commonly used materials [7]. However, these membranes have inferior mechanical properties, collapse upon wetting, and have unpredictable degradation rates [8,9]. Despite the relatively satisfactory clinical performance of these materials [10,11], the results are not fully predictable, especially in cases with higher complexity, as in furcation lesions and one- or two-wall defects [12–14]. Therefore, new techniques, such as tissue engineering, have been proposed for periodontal regeneration. Tissue engineering uses scaffolds associated with biomolecules and differentiable stem cells to form appropriate dental supportive tissues [15]. Due to the structural intricacy of the periodontal ligament and the morpho-physiological diversity of its component tissues, the design of scaffolds for periodontal regeneration is highly complex. Efforts have been made to create multiphasic scaffolds in which a specific composition and/or structure are created for each of the tissues to be generated, namely, bone, periodontal ligament, and cement [9,16]. Several new materials have been evaluated *in vitro* [17–20], but few studies have evaluated the effectiveness of these materials *in vivo* with promising results [21–23].

In addition to its structure, the composition of the scaffold is extremely important in the engineering of periodontal tissues, as it determines the biocompatibility with host tissues and stimulates regeneration or inhibition of those tissues. The scaffold’s degradation rate should be proportional to the neoformation of regenerated tissue because rapid degradation can compromise neoformation [21], whereas slow degradation can promote encapsulation [24] or bone obstruction [25]. A combination of synthetic and natural polymers, such as COL [26], has been shown to be an interesting alternative for tissue engineering, as it combines the properties of both materials [6,27]. New materials, such as copolymers containing isosorbide succinate and L-lactide moieties, have shown promising surface properties, promoting increased fibroblast adhesion and proliferation [28], and may constitute a new option for exploring the properties of cell/material interactions.

Although several alternatives have been proposed, no material is yet available to promote periodontal regeneration in an effective, consistent and predictable manner that can be easily applied in the clinic. The ideal material must simultaneously comply with the principles of GTR and tissue engineering. Although electrospun scaffolds are known to promote cell adhesion, proliferation and partial infiltration [29], cells may not completely cross the scaffold, giving rise to peripheral growth [30,31]. In the present study, one surface could block epithelial cells from crossing the scaffold, whereas the opposite surface allowed mesenchymal stem cell contact with the dental root, thus improving the cement regeneration process.

Hence, the goal of this study was to generate novel scaffolds for GTR and tissue engineering aimed at periodontal regeneration. More specifically, we combined electrospun PLLA or PisPLLA with COL, hydroxyapatite (HA) and growth factors such as bone morphogenetic protein-7 (BMP7). The membrane’s efficacy was evaluated based on *in vitro* cell proliferation and bone differentiation, whereas its *in vivo* performance was evaluated based on experimental regeneration of periodontal defects in rats.

## Materials and Methods

### Materials

PLLA (Purac, Amsterdam, the Netherlands), chloroform (Vetec, Rio de Janeiro, Brazil), dimethylformamide (Synth, Diadema, Brazil), 1,1,1,3,3,3-hexafluoro-2-propanol (Sigma-Aldrich, St. Louis, MO, USA), and HA nanoparticles (Sigma-Aldrich) were used as received. Type I COL was extracted from bovine tendon. Recombinant human BMP7 was prepared and purified at NUCEL/NETCEM (Cell and Molecular Therapy Center, University of São Paulo Medical School, São Paulo, Brazil) according to a previously published protocol [32]. Poly(isosorbide succinate) (Pis) was synthesized according to a previous study [28]. Block copolymer poly(isosorbide succinate-co-L-lactide) (PisPLLA) was synthesized in bulk via a ring-opening polycondensation reaction of L-lactide and Pis using tin(II) 2-ethylhexanoate (Sigma-Aldrich), as previously described by Casarano et al. [28]. To reach a higher molar mass, the product was subjected to chain extension using hexamethylene diisocyanate (Sigma-Aldrich). All synthetized polymers were characterized by size exclusion chromatography (SEC) in a Shimadzu CLASS-VP HPLC system (Tokyo, Japan) linked to a Shimadzu RID 10A differential refractive index detector and using four Styragel columns (10^2^, 10^3^, 10^4^, and 10^5^ A; Waters), with chloroform as the mobile phase (1 mL/min) and polystyrene as the standard (Waters) to obtain the *M*_*n*_ and *M*_*w*_ parameters. Nuclear magnetic resonance (NMR) spectroscopy of protons (^1^H) and carbon (^13^C) was performed in a Bruker DRX 500 (500 MHz and 125 MHz, respectively). The relative molar amount (*M*_*LA*_) and weight percent (%*W*_*LA*_) of L-lactide incorporated into the product were calculated based on the ^1^H-NMR spectrum according Casarano et al. [28].

### Scaffold generation and characterization

Scaffolds for cell culture were prepared by electrospinning using in-house equipment composed of a Glassman EH Series high-voltage power source and a Cole Parmer infusion pump using a syringe needle with an inner diameter of 0.584 mm as a spinning nozzle and a grounded stainless steel plate as a collecting target. The following conditions were used: a controlled room temperature of 22°C, 40% humidity, a flow rate of 4 mL/h, a distance of 18 cm between the needle and the collector, and an applied voltage of 25 kV. Electrospun mats of pure PLLA and PisPLLA were produced from 5 and 20 wt% solutions in chloroform. Scaffolds composed of polymer/COL (1:1) were obtained using a solution at a final concentration of 5 wt% in 1,1,1,3,3,3-hexafluoropropanol (HFP). Alternatively, HA nanoparticles were pre-dispersed in chloroform or HFP and added to a solution of pure polymer (PLLA or PisPLLA) or polymer/COL to result in a final composite concentration of 30% w/w. Because the COL fibers became partially soluble in aqueous media after electrospinning, scaffolds containing COL in their composition were stored in glutaraldehyde for 24 h to obtain crosslinking of the fibers. After this crosslinking treatment, the scaffolds were abundantly washed four times with a 0.02 M glycine solution for 20 min and once with deionized water to remove and neutralize the remaining glutaraldehyde.

After electrospinning, the thermal characteristics of the scaffolds were determined by differential scanning calorimetry (DSC; TA Instruments model Q10 v9.9). The specimens (5 mg) were analyzed from -50°C to 225°C at a rate of 10°C/min under 50 mL/min nitrogen flow in the first heating cycle and until 250°C in the second heating cycle.

To analyze and compare the scaffolds’ morphology, scanning electron microscopy images at 2000× magnification were obtained with a JEOL FEG 741F field emission electron microscope.

During the *in vitro* experiments, two scaffolds were also analyzed in the presence of BMP7 in the culture medium to investigate whether cell differentiation would improve, resulting in a total of ten experimental conditions (see Table 1).

**Table 1:** Material composition/experimental conditions.

Material composition/experimental condition	Abbreviation
PLLA	PLLA
PLLA and 30 wt% hydroxyapatite	PLLA/HA
PLLA and collagen (1:1)	PLLA/COL
PLLA and collagen (1:1) and 30 wt% hydroxyapatite	PLLA/COL/HA
PLLA and collagen (1:1) and 30 wt% hydroxyapatite in the presence of BMP7	PLLA/COL/HA + BMP7
PisPLLA	PisPLLA
PisPLLA and 30 wt% hydroxyapatite	PisPLLA/HA
PisPLLA and collagen (1:1)	PisPLLA/COL
PisPLLA and collagen (1:1) and 30 wt% hydroxyapatite	PisPLLA/COL/HA
PisPLLA and collagen (1:1) and 30 wt% hydroxyapatite in the presence of BMP7	PisPLLA/COL/HA + BMP7

### *In vitro* experiments

Stem cells from the dental pulp of human exfoliated deciduous teeth (SHEDs) were obtained from NUCEL/NETCEM Cell and Molecular Therapy Center, University of São Paulo Medical School, São Paulo, Brazil; by written informed consent from the person responsible for donor; this study was approved by ethics committee of University Hospital of University of São Paulo, the approval number is CEP-HU/USP 958/09 SISNEP CAAE 0075.0.198.000–09. These cells were expanded in minimum essential medium (α-MEM; Invitrogen) supplemented with 10% fetal bovine serum (FBS; Invitrogen). Depending on the test, after seeding on the different scaffolds, the cells were cultured in a basal medium composed of Dulbecco’s modified Eagle’s medium (DMEM; Invitrogen) supplemented with 10% FBS or in an osteoblastic differentiation medium (ODM) composed of DMEM, 10% FBS, 10 mM β-glycerophosphate disodium salt hydrate (Sigma-Aldrich) and 50 μg/mL L-ascorbic acid (Sigma-Aldrich). All culture media were supplemented with 1% penicillin/streptomycin (10,000 U/mL/10,000 μg/mL; Invitrogen) and 2.5 μg/mL amphotericin B (Invitrogen).

#### Cell proliferation

Cell proliferation was determined based on ^3^H-thymidine uptake into DNA. A SHED cell suspension of 2.0×10^4^ cells/well was seeded on the different scaffolds and cultured in ODM for 21 days (n = 3), at which point the cells were labeled with ^3^H-thymidine (0.037 MBq/well or 0.5 μCi/well) for 18 h. The cells were then washed twice with PBS, and 500 μL of 5% TCA was added to remove the unincorporated ^3^H-thymidine. The cells were lysed in 0.1 N NaOH and 0.1% SDS for 2 h and harvested onto glass fiber filters. Finally, the ^3^H-thymidine-labeled DNA was counted using a PerkinElmer liquid scintillation counter (Tri-Carb 2910TR; PerkinElmer). The data are expressed in counts per minute (CPM).

#### Alizarin Red staining

A SHED cell suspension (2.0×10^4^ cells/well) was seeded in culture dishes containing the different scaffolds and was cultured for 21 days in ODM (n = 3). The scaffolds were then washed with PBS (Sigma-Aldrich), fixed in 10% formaldehyde solution for 10 min and immersed in an aqueous solution with 1 vol% hydroxyl ammonium and 1 wt% Alizarin Red for 3 min. After several washes to remove excess stain, the scaffolds were dried, and the dye was desorbed from the scaffolds using 10% cetylpyridinium chloride (Sigma-Aldrich) for 1 h [33]. The absorbance of the Alizarin Red/calcium complex was measured at 570 nm for scaffolds in the presence or absence of cells. The initial mineral content of the scaffold composition was subtracted from the final mineral content.

#### Osteopontin (OPN) expression

A SHED cell suspension was prepared at 1×10^5^ cells/well and then seeded on the scaffolds and maintained in ODM for 21 days (n = 3 in two independent experiments). The cells were extracted from the scaffolds using a 0.5% trypsin solution for 40 min and were then fixed in a 1% formaldehyde solution for 10 min. The cell suspension was subsequently blocked and permeabilized in a solution of 3% FBS and 0.05% saponin in PBS. Primary antibody against OPN (63856; Abcam) at a concentration of 1:100 and secondary anti-rabbit Alexa Fluor 488 antibody (Life Technologies) at a concentration of 1:200 were used to label the cells. The cells were then washed, and the fluorescence was quantified on 10,000 cells using a FACSCalibur (BD Biosciences) with FlowJo Software (Tree Star).

#### Quantitative real-time polymerase chain reaction (qRT-PCR)

qRT-PCR experiments were performed *in vitro*. SHEDs were cultured onto the developed scaffolds or directly over conventional culture plates (control group) for 0, 7, 14 or 21 days in ODM or DMEM (n = 3). The cells were then lysed with TRIzol Reagent (Invitrogen). Total RNA was extracted according to the manufacturer’s instructions, and the quantity of RNA was assessed with a NanoDrop ND-1000 Spectrophotometer (NanoDrop Technologies). After treatment with RNase-free DNase (Invitrogen), the DNA-free RNA was used for synthesis of first-strand cDNA at 42°C for 50 min using Moloney murine leukemia virus reverse transcriptase (Invitrogen). qRT-PCR using Power SYBR Green PCR Master Mix was conducted for 45 cycles at 95°C for 15 s and at 55°C for 1 min in a 96-well format on an ABI Prism 7700 real-time PCR system (Applied Biosystems). The average Ct (threshold cycle) value for duplicate samples was used for data analysis. HMBS (hydroxymethylbilane synthase) was used to normalize the expression level of genes of interest. The primers used are shown in Table 2.

**Table 2:** List of primer sequences for the reverse (R) and (F) forward direction.

**Gene**	**Direction**	**Primer sequence**
HMBS	F	5´ TGGACCTGGTTGTTCACTCCTT 3´
	R	5´ CAACAGCATCATGAGGG 3´
RunX2	F	5´ CCATAACCGTCTTCACAAATCC 3´
	R	5´ AATGCGCCCTAAATCACTG 3´
BGP	F	5´ AAGAGACCCAGGCGCTACCT 3´
	R	5´ AACTCGTCACAGTCCGGATTG 3´
ALP	F	5´ TGTCATCATGTTCCTGGGAGAT 3´
	R	5´ TGGAGCTGACCCTTGAGGAT 3´

### *In vivo* regeneration

The experiment was approved by the ethics committee for animal use (CEUA) of the Institute of Chemistry of the University of São Paulo (approval number 17/2012). Twenty three-month-old Wistar rats were subjected to intraperitoneal anesthesia (ketamine 10 mg/Kg and xylazine 3.3 mg/Kg). An extra oral incision was applied in the mandibular base, the masseter ligament in the inferior border was cut, and the muscle was separated from the bone to expose the mandibular bone. A periodontal fenestration defect was created at the vestibular root of the first molar using a carbide burn (n° 4) under irrigation. The defect dimensions were standardized at a 5 mm length and a 1.5 mm width according to a previous study [34,35]. The surgery was performed using a surgical microscope (Olympus), and the defect was deepened until reaching the periodontium, which was clinically characterized by bleeding and root exposure. A scaffold with or without cells was placed over the defect, the masseter muscle was replaced in its original position, and the incision was sutured with 6.0 nylon wire. Thirty days after the surgery, the animals were sacrificed under CO_2_. The jaws were fixed in 0.1% glutaraldehyde (Sigma-Aldrich) and 4% formaldehyde (Sigma-Aldrich) in 0.1 M cacodylate buffer (Sigma-Aldrich), pH 7.4, and decalcified in a 4.13% EDTA (Sigma-Aldrich) aqueous solution for 40 days. Pieces were then embedded in paraffin, and histological slices were prepared and stained with hematoxylin/eosin. Five experimental groups were evaluated: a control group with no material, PLLA/COL/HA matrix with or without seeded SHEDs, and PisPLLA/COL/HA matrix with or without SHEDs. In the groups in which scaffolds containing cells were implanted in the defect, a total of 1.0×10^6^ cells were seeded on each scaffold 24 h before implantation and were maintained in culture in DMEM.

The histological images were obtained on an Olympus Light Microscope (BX60 F-3, Japan) with magnification of 40 and 200x. The images with magnification of 40x (n = 6 per group) were used to histomorphometric analysis. The periodontal ligament thicknesses were measured perpendicular to teeth long axis, from cementum to alveolar bone in intervals around 100 μm, within the area between the defect edges. The bone neoformation area (immature bone) was measured between the edges of the defect. Both the measured were performed using the software Image J 1.48v (Wayne Rasband National Institute of Health, USA).

### Statistical analysis

For each test, the data were subjected to one-way ANOVA and Tukey’s test, with a global level of significance of 95% (α = 0.05) after confirming the homoscedasticity (Levene’s test) and normality (Anderson-Darling test) of the data. The comparison among mean values is represented using lettering, with groups sharing at least one similar letter presenting no statistically significant difference (p≥0.05) and groups with all different letters presenting a significant difference (p<0.05).

## Results and Discussion

### Scaffold generation and characterization

To test the ability of PisPLLA containing different combinations of COL and/or HA nanoparticles to generate an environment adequate for bone and periodontal regeneration, eight different scaffolds were electrospun and tested: four were based on PLLA, a well-known and widely used polymer in tissue engineering that was used here as a standard, and the other four were based on PisPLLA, a new material originating from co-polymerization of L-lactide and isosorbide succinate that has been presented as a promising scaffold in regard to fibroblast adhesion and proliferation [28]. COL and/or HA were added to these polymers in an attempt to mimic the extracellular matrix and to generate an adequate environment for bone and periodontal regeneration. HA was included as a composite, with a load up to 30 wt%, because several studies have already reported its potential to stimulate osteogenesis and tissue mineralization [36,37]. COL was used at a 1:1 ratio with the polyester (PLLA or PisPLLA) to enable a stronger interaction between the material and the cells and also to more greatly stimulate bone cell differentiation [38]. A previous study indicated that blended PLLA/COL electrospun scaffolds at a 1:1 ratio, as used in the present study, are able to generate high tensile strength and a high Young’s modulus and can promote bone cell differentiation, even in the absence of osteogenic medium [27]. In the present study, the morphological and thermal characteristics of all eight scaffolds were determined, and the scaffolds were subjected to proliferation and bone differentiation assays *in vitro*. The two materials in which the polymers (PLLA or PisPLLA) were concomitantly associated with COL and HA were also evaluated in cell experiments with the addition of the growth factor BMP7 in the culture medium. This growth factor was selected due to its previously proven efficacy in stimulating bone regeneration [39]. This growth factor was directly added to the culture to observe whether it could improve upon the results obtained with the scaffolds only or whether the scaffold materials alone would be enough to influence cell differentiation.

**Fig 1:**
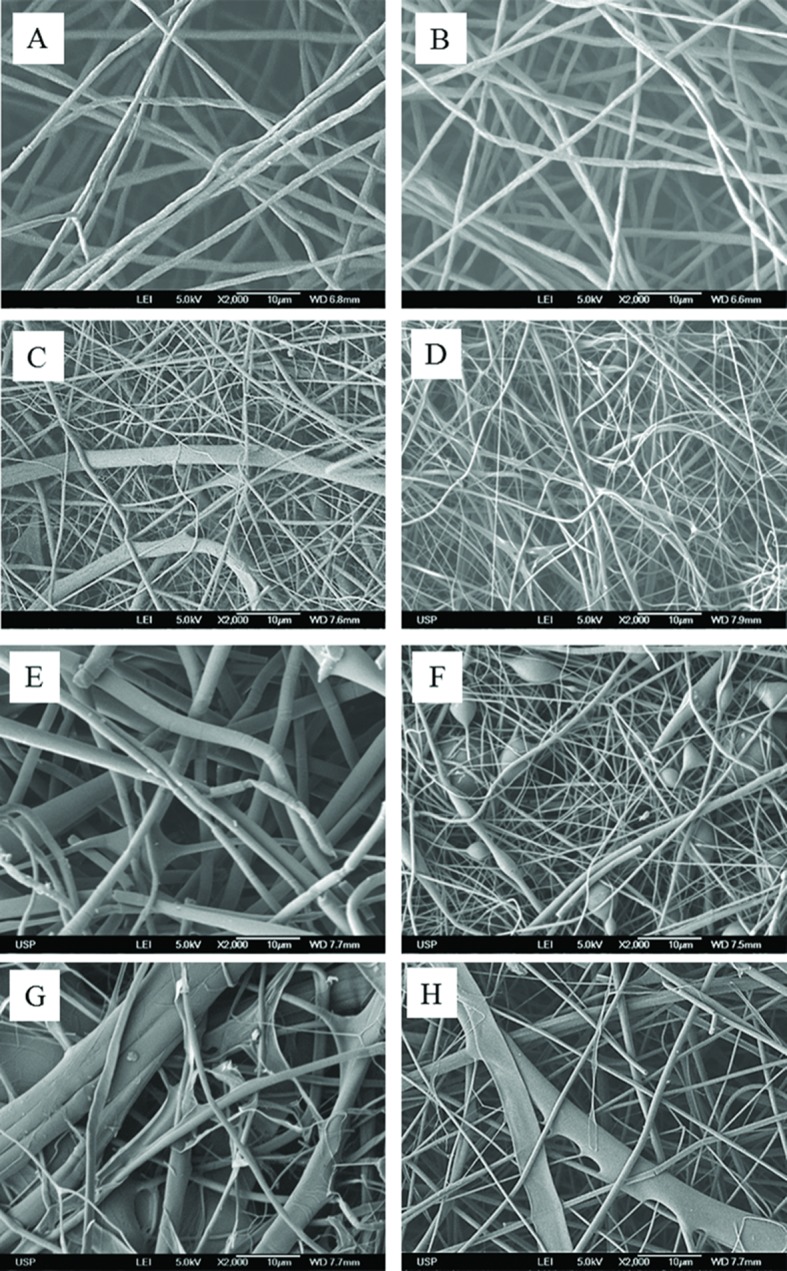
Scaffold morphology. Scanning electron micrograph of electrospun scaffolds composed of (A) PLLA, (B) PLLA/HA, (C) PLLA/COL, (D) PLLA/COL/HA, (E) PisPLLA, (F) PisPLLA/HA, (G) PisPLLA/COL, or (H) PisPLLA/COL/HA.

PisPLLA was synthetized by a copolymerization reaction of L-lactide with a pre-polymer of Pis, as previously described by Casarano et al. [28]. After the chain extension, PisPLLA was obtained with an *M*_*n*_ and *M*_*w*_ of 36.0 and 37.1 kD, respectively. Although these values are slightly lower than previously reported values (56 and 130 kD for *M*_*n*_ and *M*_*w*_, respectively [28]), these results were considered to be appropriate for this study, as they yielded electrospun mats of the same morphological quality, as described below (Fig 1).

The ^1^H-NMR and ^13^C-NMR (S1 Fig) spectra supported the expected structure because they revealed the same absorption signals as previously published for the material [28]. A ^13^C-NMR signal at 169.6 corresponds to L-lactide carbonyl moiety absorption, whereas signals at 171.2 and 171.5 ppm correspond to the same moiety from the succinate monomer, indicating the block constitution of the polymer [28]. Additionally, the integration of ^1^H-NMR signals at 1.58 and 4.82 ppm was used to calculate the relative weight percent of each monomer in the copolymer, as described by Casarano et al. [28]. Again, the value found, namely, 72% of L-lactide (against 28% of isosorbide succinate), was very close to the expected number related to the feed (75% of L-lactide) and similar to the 74% found by Casarano et al.[28].

Regarding the degradation rate, previous studies from our group (Casarano, R. and Catalani, L.H., unpublished results) showed very similar rates of degradation for PLLA and PisPLLA of different grades when subjected to acidic and basic conditions (i.e., pH 2 and pH 12 solutions, respectively). Additionally, all materials used maintained their structure after 22 days in culture media.

Fig 1 shows a scanning electron micrograph of the PLLA- and PisPLLA-based scaffolds. Scaffolds composed of pure PLLA or PisPLLA showed fibers with homogeneous diameters (Fig 1A and 1E, respectively), but the PisPLLA showed a higher mean diameter than the PLLA did (1.87±0.9^a^ μm and 1.12±0.2^bc^ μm, respectively; see S2 Fig).

For both polymers, the mats containing COL were composed of fibers of heterogeneous calibers and shapes (Fig 1C, 1D, 1G and 1H). A similar effect was previously described following electrospinning of co-solutions of PET/COL [40]. In that case, the large distribution of diameters was interpreted as the result of phase separation during spinning, giving rise to a hybrid mat composed of fibers rich in either polyester or COL. Finally, incorporation of HA nanoparticles into the PLLA scaffold (Fig 1B) did not alter the fiber diameter (PLLA, 1.12±0.2 μm; PLLA/HA, 1.06±0.4 μm; S2 Fig), whereas incorporation into PisPLLA significantly reduced the fiber diameter (PisPLLA, 1.87±0.9 μm; PisPLLA/HA, 0.2±0.1 μm). There were areas of fiber thickening in the material, but such a pattern was not observed when PisPLLA was associated with COL and HA (Fig 1H).

The results of thermal analysis of the electrospun scaffolds, as obtained by DSC, are shown in Table 3. As a general observation, the two groups of materials (based on PLLA and PisPLLA) presented similar glass-transition temperature (T_g_) values, as has also been observed by Casarano et al. [28]. However, the PisPLLA-based materials showed a consistently lower melting temperature (T_m_), or ΔT = 18.4 to 23.4°C, relative to the PLLA-based materials, as shown in Table 3. In fact, a previous study reported a reduction in the melting temperature, from 13 to 20°C, for several PisPLLA scaffolds relative to PLLA scaffolds [28]. The same study highlighted that PisPLLA is an amorphous polymer, whereas PLLA is semi-crystalline [28]. Therefore, it was expected that PisPLLA-based materials would present crystallinity and would melt at lower temperatures than materials based on PLLA would. The scaffold composed of pure PLLA was the only material that developed recrystallization during the heating cycle (Table 3). During electrospinning, due to rapid polymer stretching and deposition over the collector, a loss of polymer crystallinity occurs. As a result, that thermal treatment causes molecular rearrangement of the fibers, thereby increasing the material crystallinity [41]. However, when HA, COL or Pis is associated with PLLA, this association interferes with the recrystallization process.

**Table 3:** The individual glass-transition temperature (T_g_), crystallization temperature (T_c_), melting temperature (T_m_), heat of fusion (ΔH_f_) and crystallinity (X_c_) of the studied materials.

Material	T_g_ (°C)	T_c_ (°C)	T_m_ (°C)	ΔH_f_ (J/g)	Χ_c_ (%)
PLLA	61.2±0.9	106.7±0.8	180.3±0.8	46.2±2.5	41.1±1.1
PLLA/HA	61.3±0.9	-	180.5±1.1	31.4±6.1	33.7±6.5
PLLA/COL	58.3±0.3	-	176.2±2.7	17.4±1.4	18.7±1.5
PLLA/COL/HA	56.2±1.2	-	178.6±1.1	13.7±1.5	14.7±1.6
PisPLLA	62.0±0.8	-	160.8±0.4	19.3±0.4	20.7±0.5
PisPLLA/HA	62.0±1.7	-	157.1±0.5	11.6±1.2	12.4±1.3
PisPLLA/COL	56.3±0.2	-	157.7±0.5	4.9±0.9	5.2±1.0
PisPLLA/COL/HA	59.0±1.3	-	160.2±2.0	8.2±0.8	8.8±0.9

### Evaluation of SHED proliferation in the scaffolds

**Fig 2:**
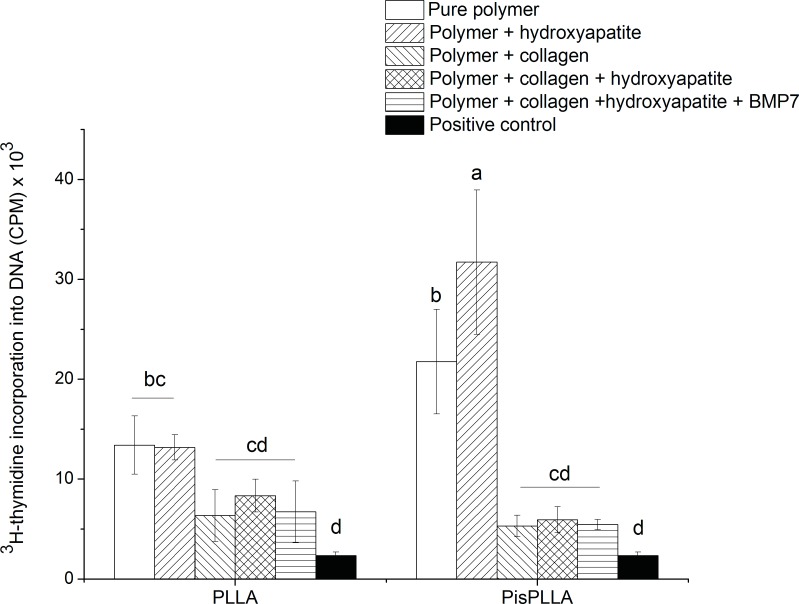
Effect of PLLA or PisPLLA on SHED cell proliferation. SHEDs were cultured in DMEM for 21 days, and their proliferation was assessed by ^3^H-thymidine incorporation measurement. The results are expressed as the mean ± s.d. (n = 3). Similar letters indicate no significant difference.

The evaluated scaffolds differed with respect to fiber diameter, porosity and crystallinity. In addition, the different compositions yielded materials with different hydrophilicities and required use of a potentially toxic agent, such as glutaraldehyde, to crosslink the fibers. As all of these factors can affect cell adhesion and growth, proliferation assays were performed to test the scaffolds’ ability to maintain stem cell division up to 21 days in culture in the presence of bone differentiation medium. It is known that at 21 days of culture, cell proliferation reaches a plateau phase, and bone differentiation is predominant, with extracellular matrix mineralization [33]. This time-point was selected only to evaluate the cell viability in this final stage of maturation. SHED proliferation, as measured by thymidine incorporation at 21 days of culture (Fig 2), was higher for pure PisPLLA and PisPLLA/HA scaffolds and very similar for the other scaffolds compared with PLLA alone. The capacity of PisPLLA with or without HA to improve cell adhesion and proliferation relative to those noted for PLLA has previously been observed in fibroblast culture [28]. It is believed that the higher superficial energy (54 mN/m) of PisPLLA compared with PLLA (40 mN/m) [28] is in the optimal range of hydrophilicity that favors wettability, dispersion, adhesion, and cell proliferation [42]. However, the integration of COL into the PisPLLA-based materials likely increased the hydrophilicity beyond this optimal range, consequently resulting in a lower cell proliferation rate than that observed for the other PisPLLA-based materials (Fig 2).

BMP7 was added to the cell medium to evaluate whether its presence would alter the original effect observed in the *in vitro* studies. As BMP7 stimulates cell differentiation, we expected no effect for BMP7 in the proliferation assay, as shown in Fig 2, because the differentiation stimulus should reduce/inhibit the cell division stimulus [43,44]. The activity of the BMP7 was previously examined in a culture of C2C12 cells using an alkaline phosphatase (ALP) assay, as previous published [32].

### Evaluation of potential for bone differentiation

As the scaffolds were designed to be used in bone and periodontal tissue regeneration, evaluation of not only cell adhesion and proliferation but also bone differentiation capacity was necessary. Therefore, the experiments below were carried out to map the influence of the materials on SHED differentiation into bone cells and on the maturation of the bone extracellular matrix.

#### Alizarin Red assay

**Fig 3:**
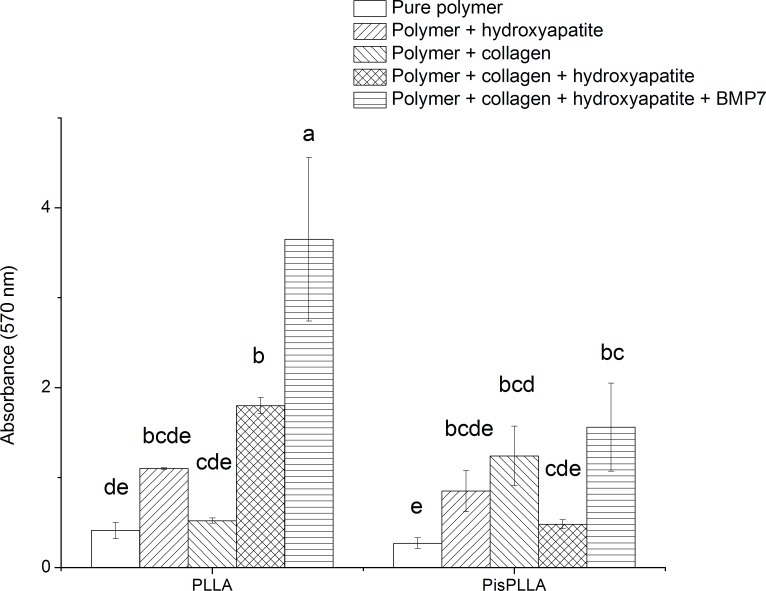
SHED cells stained with Alizarin Red. SHEDs were cultured on PLLA- or PisPLLA-based materials and stained with Alizarin Red at day 21. The values are the mean ± s.d. (n = 3). Similar letters indicate no significant difference.

The Alizarin Red assay relies on the binding of dye to the calcium phosphate crystals present in the extracellular matrix, which are an indicator of an advanced stage of osteoblastic maturation [33]. In the present study, the absorbance of the Alizarin Red/calcium complex (Fig 3) was normalized by the number of cells/mm^2^ observed by confocal microscopy (based on DAPI-stained nuclei) in the scaffold (data not shown). Regarding the PLLA-based materials, PLLA/COL/HA displayed higher mineral deposition than the pure PLLA or PLLA/COL (Fig 3), even after subtraction of the initial mineral content of the HA-containing scaffold from the final mineral content, as described previously. These results are in agreement with a previous study in which a synergistic effect of COL and HA was observed when both were associated with PLLA, thus increasing bone cell differentiation and matrix mineralization [37]. The degree of mineralization of the PisPLLA scaffolds in the present study was similar to that observed for all of the materials except for PLLA (Fig 3), indicating that association with COL and HA did not result in increased mineralization. Additionally, the mineralization of PisPLLA/COL/HA was lower than that of PLLA/COL/HA (Fig 3).

It was expected that addition of both bone bio-modulators, namely, HA and COL, would provide higher levels of mineralization of the extracellular matrix relative to the levels in the materials with only one or neither of the bio-modulators. This phenomenon was observed for PLLA, but not for the PisPLLA materials. We speculated that the higher crystallinity and rigidity of PLLA favored the synergistic effect of COL and HA with respect to scaffold mineralization.

Additionally, the inclusion of BMP7 in the culture medium of the two types of materials, namely, PLLA/COL/HA and PisPLLA/COL/HA, increased mineralization of the former, but not the latter (Fig 3). Several studies have shown the capacity of BMPs to improve bone cell differentiation *in vitro* and *in vivo* [32,39]. However, in the PisPLLA materials, the effect of BMP7 was limited.

#### Flow cytometry analysis of labeled OPN

OPN is a highly phosphorylated bone protein that is part of the bone extracellular matrix. This protein displays poly-aspartic acid, which is able to bind to HA and RGD areas, which are in turn able to bind to cells. Therefore, OPN functions as a bridge between cells and the inorganic content of the bone [45]. OPN has been adopted as a marker of mature osteoblasts; its peak expression has been observed at both 7 and 21 days of differentiation, depending on the culture conditions and cell lineage [33,46]. An *in vitro* study has demonstrated that even though OPN is an extracellular matrix protein, in culture, this protein accumulates in the cytoplasm, allowing its detection and measurement by flow cytometry, as performed in the present study [46].

**Fig 4:**
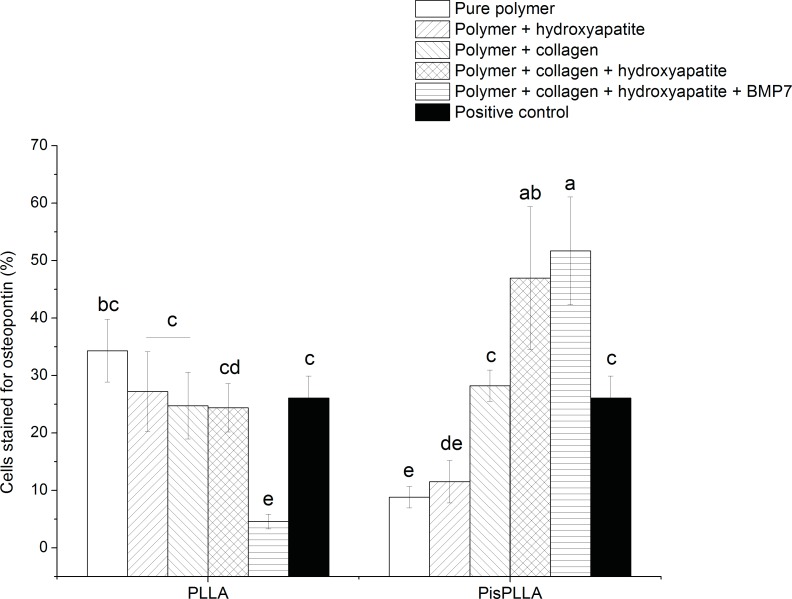
Effects of PLLA and PisPLLA on OPN protein expression. FACS analysis was performed after 21 days of culture. Staining for isotype controls was included in all experiments (mean ± s.d.; n = 6). Similar letters indicate no significant difference.

When comparing the OPN expression for the eight scaffolds evaluated (Fig 4), we observed that the PisPLLA/COL/HA material displayed a higher level of OPN labeling, indicating greater cell differentiation promoted by this scaffold. The other materials presented similar and intermediate values. Association with COL and HA had a synergistic effect, leading to increased protein expression. This effect was similar to the effect previously observed for PLLA with respect to the mineralization process (Fig 4). Association with COL and HA creates conditions favorable for cell differentiation, particularly when associated with PisPLLA. The coadjuvant effect of BMP7 was negligible for PisPLLA/COL/HA, but interestingly, the presence of BMP reduced the number of cells expressing OPN (Fig 4). This growth factor most likely increases OPN release from the PLLA/COL/HA material and into the extracellular environment. The presence of a higher amount of OPN in the extracellular matrix can help to explain the higher mineralization of the extracellular matrix observed in Fig 3, as previously discussed.

#### Analysis of gene expression by qRT-PCR

Given that PLLA/COL/HA demonstrated an ability to improve mineralization, as assessed by Alizarin Red assay (Fig 3), and that PisPLLA/COL/HA demonstrated increased OPN expression, as assessed by flow cytometry (Fig 4), both materials were selected to be evaluated by *in vitro* qRT-PCR experiments.

Analysis of gene expression by qRT-PCR was performed for three genetic markers of bone differentiation, namely, RunX2, ALP and osteocalcin (bone Gla protein, or BGP), at 0, 7, 14 and 21 days of culture in ODM or DMEM. The culture in ODM revealed the osteoconductive properties of the scaffolds, whereas the culture in DMEM evidenced the osteoinductive capacity of the materials.

**Fig 5:**
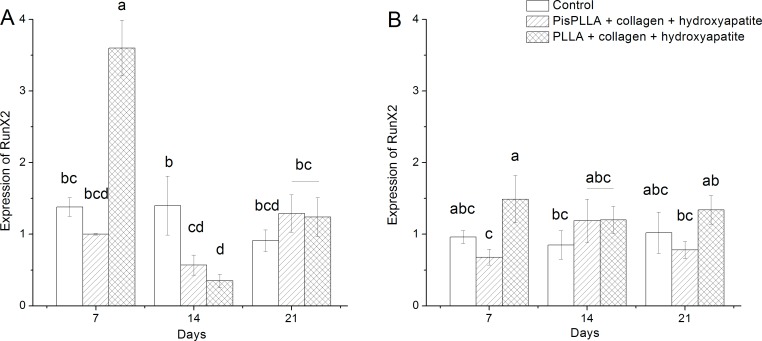
RunX2 levels of SHED cells in PLLA and PisPLLA scaffolds. Relative expression of the RunX2 gene in SHEDs cultured in osteogenic medium (A) or basal medium (B). The dotted line shows the expression in undifferentiated SHEDs. HMBS was used as an internal control. The values are the mean ± s.d. (n = 3). Similar letters indicate no significant difference.

RunX2 is the main transcription factor required for bone differentiation, as it is involved in all phases of bone maturation [47]. In general, we observed only one peak of RunX2 expression, specifically at 7 days of culture in the presence of ODM and the PLLA/COL/HA material (Fig 5A), indicating a higher propensity of this material for osteoconduction. RUNX2 expression was similar following culture in DMEM in the presence of all other materials and for all periods of time studied (Fig 5B), with little variation relative to the basal levels. These results are in agreement with previous studies [48,49] that showed that RunX2 is weakly expressed under basal culture conditions.

**Fig 6:**
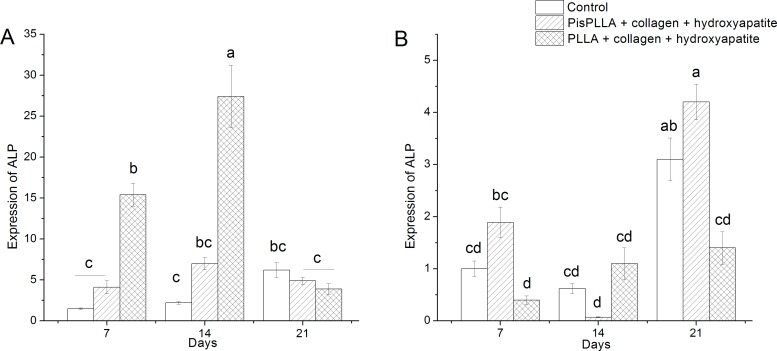
ALP levels of SHED cells in PLLA and PisPLLA scaffolds. Relative expression of the ALP gene in SHEDs cultured in osteogenic medium (A) or basal medium (B). The dotted line shows the expression in undifferentiated SHEDs. HMBS was used as an internal control. The values are the mean ± s.d. (n = 3). Similar letters indicate no significant difference.

The ALP enzyme is secreted at the initial stages of bone differentiation [33], displaying its expression peak later than that of RunX2 but earlier than those of other proteins of extracellular matrix, such as OPN or osteocalcin. Under conditions of osteogenic culture (Fig 6A) ALP expression peaked at 14 days of culture for PLLA/COL/HA, whereas for PisPLLA/COL/HA and control culture, ALP expression was similar at all time intervals observed, indicating the potential of the PLLA/COL/HA matrix to support bone cell differentiation. Schofer et al. have also observed that in ODM, the ALP expression for a PLLA/COL blend peaked at 10 days of culture, and decreased ALP expression was noted at 22 days of culture [50]. In the current study, the PLLA/COL/HA material displayed higher ALP expression at 7 and 14 days (Fig 6A), with approximately three times higher expression than that observed for the PisPLLA/COL/HA material, which indicates higher osteoconductivity capacity; in contrast, the control group showed the lowest values. At 21 days, with the increase in osteoblastic maturation in the materials, a similar ALP expression level was found in all of the groups (Fig 6A).

ALP expression in DMEM (Fig 6B) was lower and slower than that observed in ODM under all conditions, with the ALP expression peak occurring at 21 days under all conditions. Higher ALP expression was observed for the PisPLLA/COL/HA material at 21 days (Fig 6B), indicating that it did not inhibit the endogenous cell differentiation compared to PLLA/COL/HA at this timeframe. Although ALP expression in the PLLA/COL/HA material was higher than the basal level, it was still lower than the level in the control group at 21 days, indicating an inhibitory effect on ALP expression by this material (Fig 6B).

**Fig 7:**
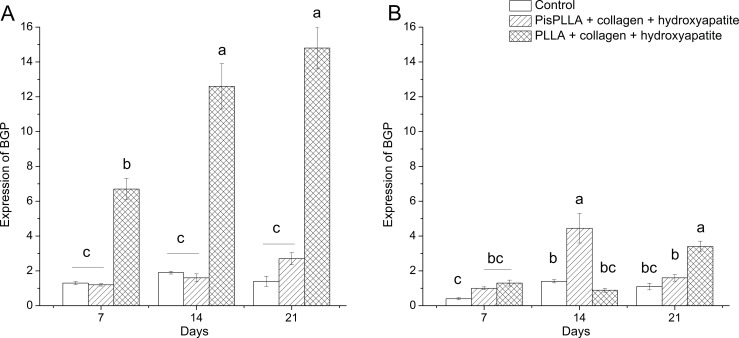
BGP levels of SHED cells in PLLA and PisPLLA scaffolds. Relative expression of the BGP gene in SHEDs cultured in osteogenic medium (A) or basal medium (B). The dotted line shows the expression in undifferentiated SHEDs. HMBS was used as an internal control. The values are the mean ± s.d. (n = 3). Similar letters indicate no significant difference.

Finally, the BGP gene encodes osteocalcin, an extracellular matrix protein [51] important for matrix mineralization that is expressed during the late stages of differentiation [52]. As observed for ALP, BGP expression in ODM (Fig 7A) was higher in the PLLA/COL/HA material at all time-points, with 5 to 6 times higher expression than that observed for the other materials, again highlighting the osteoconductive potential of this material. In agreement with the present study, other authors have reported an osteocalcin expression peak at 21 days of culture [33,50]. A discrete increase in BGP expression as a function of time was also observed for the PisPLLA/COL/HA, but without a significant difference relative to the control group, indicating the weak influence of this material during ODM culture (Fig 7A). However, under basal conditions (Fig 7B), the PisPLLA/COL/HA showed earlier BGP expression at 14 days, whereas, PLLA/COL/HA supported the BGP expression at 21 days point. Both were higher than control group at 14 and 21 days respectively, confirming the osteoinductive potential of the matrices. Based on the gene expression analysis of the three bone markers studied, it can be concluded that under osteogenic culture conditions, the PLLA/COL/HA material is preferable due to its osteoconductive properties (Figs 5A, 6A and 7A).

Under basal culture conditions, both materials, namely, PLLA/COL/HA and PisPLLA/COL/HA, displayed a relative capacity of osteodifferentiation (Figs 6B and 7B). However, the PisPLLA/COL/HA material presented faster expression of BGP gene. The similarity of the response of PisPLLA/COL/HA with control group in relation to ALP expression and the decrease of ALP expression on PLLA/COL/HA compared to control group at 21 days indicate that the former is able to maintain the differentiation process while the PLLA/COL/HA presents some inhibitory effect.

### Histological analysis

**Fig 8:**
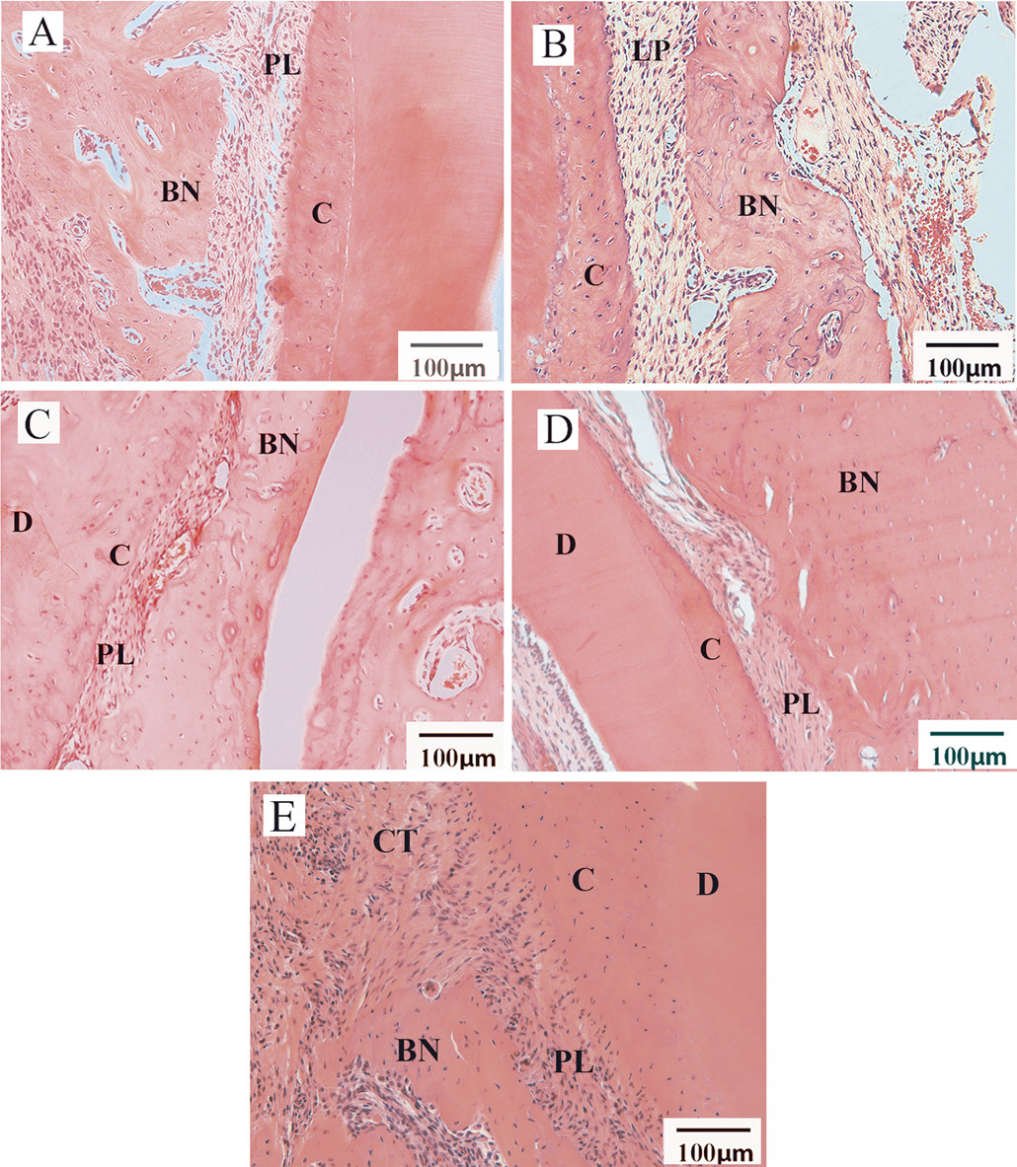
Histological analysis of periodontal defect 30 days after surgery (n = 6 per group). Image A indicates the use of PLLA/COL/HA membranes. Image B shows PLLA/COL/HA associated with SHEDs. Image C shows PisPLLA/COL/HA membranes. Image D shows PisPLLA/COL/HA membranes associated with SHEDs. Image E indicates the negative control group. New bone formations (BN), periodontal ligament (PL), cementum (C), dentin (D) and conjunctive tissue (CT) are indicated in the figures.

The *in vivo* study was performed with six animals for each of the following experimental conditions: PisPLLA/COL/HA and PLLA/COL/HA membranes; respectively, the most promising PisPLLA condition and its PLLA counterpart reference. Previous *in vivo* studies from our laboratory (unpublished results) demonstrated that PisPLLA alone or in combination with HA presented only modest bone regeneration as compared to PLLA. The histological analysis was based on the pattern observed in each group, and representative images for each condition are shown in Fig 8. The histomorphometric analysis is presented on Table 4. The power of the test was calculated as 89% (Minitab® statistical software). In general, we observed similar new bone formation areas between the PLLA/COL/HA and PisPLLA/COL/HA membranes, with levels higher than those in the control group. Additionally, the presence of SHEDs did not introduce an advantage (Table 4). Regarding the thickness of the new periodontal ligament generated, the histomorphometric analysis showed similar values for all of the groups evaluated, and these results were also similar to the thickness of physiological periodontal ligament (Table 4).

**Table 4:** New bone formation area and periodontal ligament thickness for the studied materials.

Material	New bone formation area (mm^2^)*	Periodontal ligament thickness (mm)*
PLLA/COL/HA	0.28±0.09 ^a^	0.099±0.02 ^a^
PisPLLA/COL/HA	0.28±0.09 ^a^	0.125±0.01 ^a^
PLLA/COL/HA + SHEDs	0.27±0.09 ^a^	0.109±0.04 ^a^
PisPLLA/COL/HA + SHEDs	0.22±0.07 ^ab^	0.118±0.04 ^a^
Control	0.06±0.01^b^	0.100±0.03 ^a^
Physiological periodontal ligament	-	0.110±0.02 ^a^

*Superscript letters refers to statistical analysis where similar letters indicate no significant difference.

According the histological analysis (S3 Fig), we observed total closure of the periodontal defect in the group with PLLA/COL/HA without cells. The well-defined edges of the defect could be observed to abut new bone formation of large volume and high thickness in the region. Additionally, we observed increased thickness of the cellular cementum, with an adequate ligament space but unaligned COL fibers (Fig 8A and S3 Fig). In certain slices, the scaffolds were observed to be practically intact in the defect region. The PisPLLA/COL/HA scaffolds without cells showed new bone formation, with total closure of the defect and a new bone formation area similar to that observed for PLLA/COL/HA (Fig 8C, S3 Fig and Table 4). However, the cementum layer did not become thicker in all of the animals, and the periodontal ligament showed certain areas with well-aligned COL fibers (Fig 8C). Other areas showed that the periodontal space was slightly reduced, but no zones of ankylosis were present. The PisPLLA/COL/HA was degraded faster than the PLLA was *in vivo*, and remains of the degraded scaffolds surrounded by multinucleated cells were observed in one animal, indicating a foreign-body reaction to this material. Furthermore, in certain animals, it was possible to observe small areas of dentin and/or cementum resorption, which could have been triggered by certain byproducts of PisPLLA/COL/HA degradation (S3 Fig). When associated with SHEDs before implantation, both materials also presented bone repair, but the new bone formation was not improved (Fig 8B and 8D). A similar area of new bone formation was noted as for the materials without cells (Table 4). Nevertheless, in certain animals, partial closure of the defect or complete closure, although with a thinner bone layer over the defect, was observed (S3 Fig). The other aspects of bone regeneration were similar to those in the respective materials without cells (Fig 8B and 8D).

Finally, in the control group, there was marginal new bone formation at the edges of the defect, but it was not closed, and the area was more limited than in the other groups (S3 Fig and Table 4). Although an increase in the cementum thickness occurred, it was followed by a decrease in the ligament space.

For both materials studied, the presence of cells inhibited bone regeneration, possibly due to changes in the cell signaling and chemotaxis in the environment being more effective for periodontal regeneration than the use of a membrane alone, such as in GTR. This result agrees with a study that evaluated bone and periosteal regeneration in rabbit jaws and did not observe the influence of bone marrow mesenchymal stem cells on tissue regeneration [53].

The PisPLLA/COL/HA displayed rapid degradation *in vivo*, with only pieces of the scaffolds being found after four weeks, whereas the PLLA/COL/HA maintained its structure during this interval of time. Studies in animals have demonstrated that a membrane should be in position for at least four to six weeks to guarantee periodontal regeneration [8,9], which agrees with the better periodontal regeneration results observed here for the PLLA/COL/HA material. Commercial and experimental COL membranes, such as PisPLLA/COL/HA membranes, have also shown a rapid resorption rate of 6 to 38 days *in vivo* [8,54,55]. The rapid and unpredictable absorption rate of COL membranes has limited the clinical success of these materials [9,55]. The faster degradation rate of PisPLLA material also points to its potential in drug delivery applications.

Regarding bone regeneration, both PLLA/COL/HA and PisPLLA/COL/HA were able to promote new bone formation in a similar manner, with complete closure of the defect and an absence of resorption. Therefore, either of these materials can be used as membranes in guided bone regeneration, depending on the degradation rate desired for the clinical condition.

## Conclusions

The purpose of this study was to develop a new scaffold based on PisPLLA as an alternative material, with the aim of fostering bone and periodontal regeneration. We also evaluated the efficacy of COL and HA inclusion to this material and to PLLA. We showed that PisPLLA associated with both COL and HA improved the differentiation and maturation of bone cells (OPN assay) while the PLLA/COL/HA improved the mineralization of extracellular matrix (alizarin red assay). Additionally, according to qRT-PCR results (Fig 7B), PisPLLA/COL/HA induced stem cells to express BGP during culture in basal medium at 14 days, earlier than PLLA/COL/HA, reaching this expression at 21 days. Also, the PisPLLA/COL/HA material did not inhibit the expression of ALP, maintaining the endogenous cell differentiation in basal conditions. Finally, the PisPLLA/COL/HA material, even in the absence of stem cells, was able to promote bone and periodontal regeneration in our *in vivo* study. Therefore, this new membrane is a promising material for application in GTR and tissue engineering.

Furthermore, PLLA/COL/HA presented high potential for osteoconduction, for mineralization of the extracellular matrix, and for expression of BGP even in basal conditions. Comparable performance was observed for the two materials in terms of new bone and periodontal formation *in vivo*. The main difference observed between the materials was the degradation rate, which was slower for the PLLA-based material.

## Supporting Information

S1 Fig ^1^H-NMR (A) and ^13^C-NMR analyses. Spectra of ^1^H-NMR (A) and ^13^C-NMR (B) obtained for the PisPLLA polymer. In B, the number 1 indicates the hydrogen peak relative to L-lactide, and the number 2 indicates the hydrogen peak relative to Pis.
(TIF)

S2 Fig Fiber diameters. Fiber diameter distributions for scaffolds composed of (A) PLLA, (B) PLLA/HA, (C) PLLA/COL, (D) PLLA/COL/HA, (E) PisPLLA, (F) PisPLLA/HA, (G) PisPLLA/COL or (H) PisPLLA/COL/HA. Similar letters indicate no significant difference.
(TIF)

S3 Fig Histological analysis of periodontal defect 30 days after surgery. Images A and B indicate the use of PLLA/COL/HA membranes. Images C and D show PLLA/COL/HA associated with SHEDs. Images E and F show PisPLLA/COL/HA membranes. Images G and H show PisPLLA/COL/HA membranes associated with SHEDs. Images I and J show the negative control, without the use of the materials or cells. In images A, C, E, G and I the insets show the edges of the periodontal defect. New bone formation (BN), periodontal ligament (PL), cementum (C), dentin (D), conjunctive tissue (CT), scaffolds (SC), and root resorption (RR) are indicated in the figures.
(TIF)
