# Supplementary figures and images for: Combination of Bioactive Polymeric Membranes and Stem Cells for Periodontal Regeneration: In Vitro and In Vivo Analyses
Source: PLoS One. 2016 Mar 31;11(3):e0152412. doi: 10.1371/journal.pone.0152412 (PMC4816539; doi:10.1371/journal.pone.0152412)

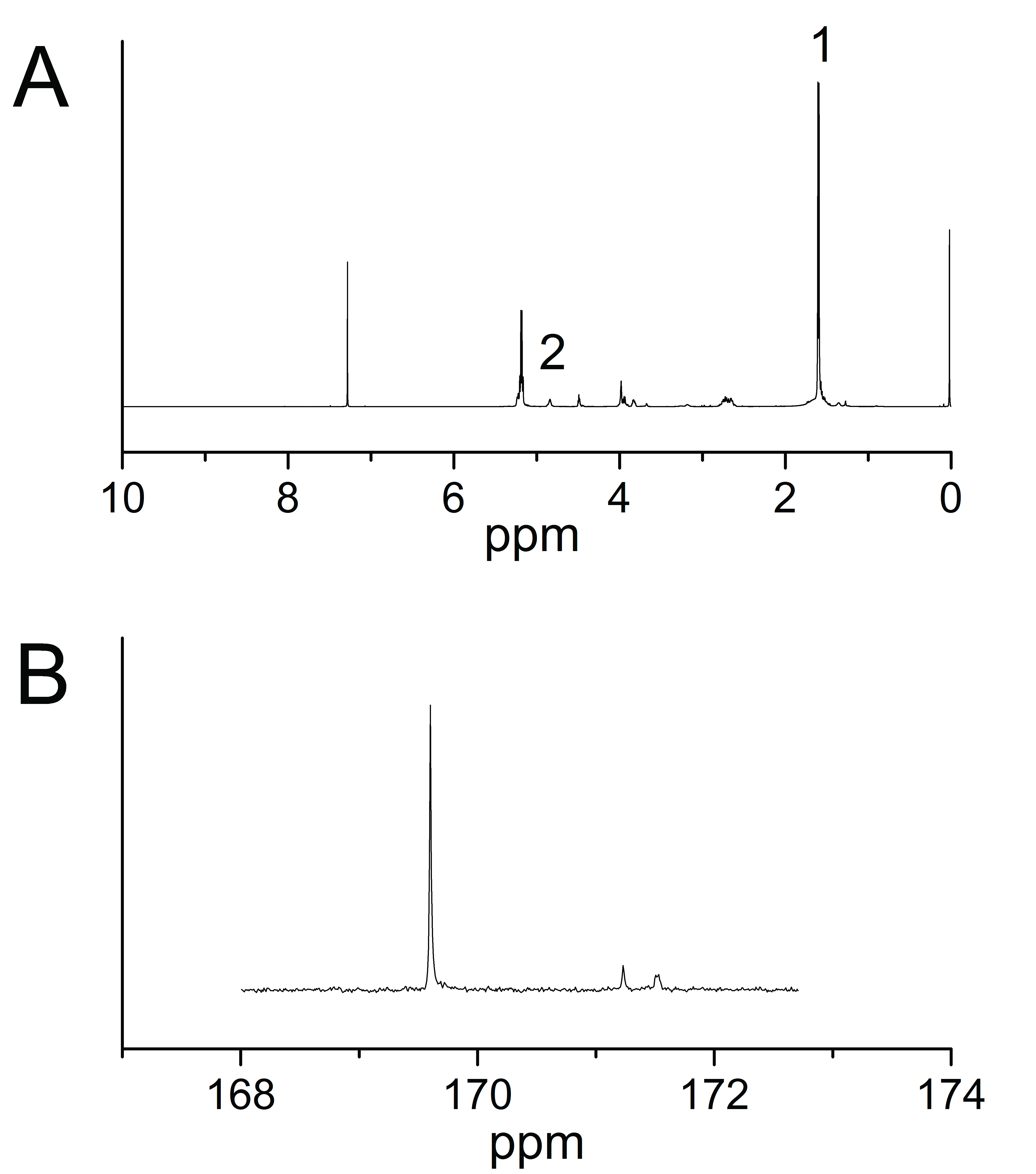

Supplement: S1 Fig — Spectra of 1H-NMR (A) and 13C-NMR (B) obtained for the PisPLLA polymer. In B, the number 1 indicates the hydrogen peak relative to L-lactide, and the number 2 indicates the hydrogen peak relative to Pis. (TIF) [file pone.0152412.s001.tif]

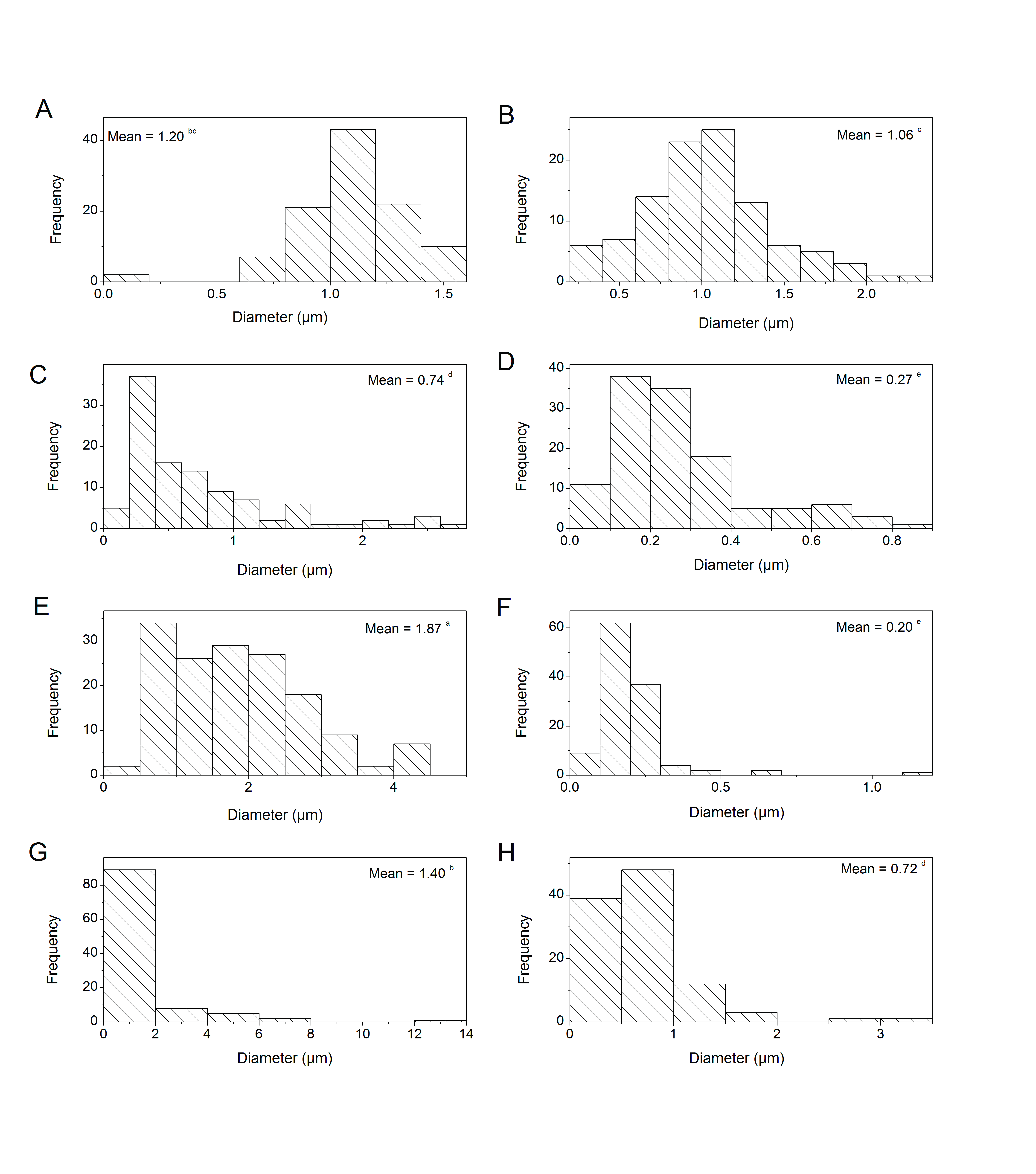

Supplement: S2 Fig — Fiber diameter distributions for scaffolds composed of (A) PLLA, (B) PLLA/HA, (C) PLLA/COL, (D) PLLA/COL/HA, (E) PisPLLA, (F) PisPLLA/HA, (G) PisPLLA/COL or (H) PisPLLA/COL/HA. Similar letters indicate no significant difference. (TIF) [file pone.0152412.s002.tif]

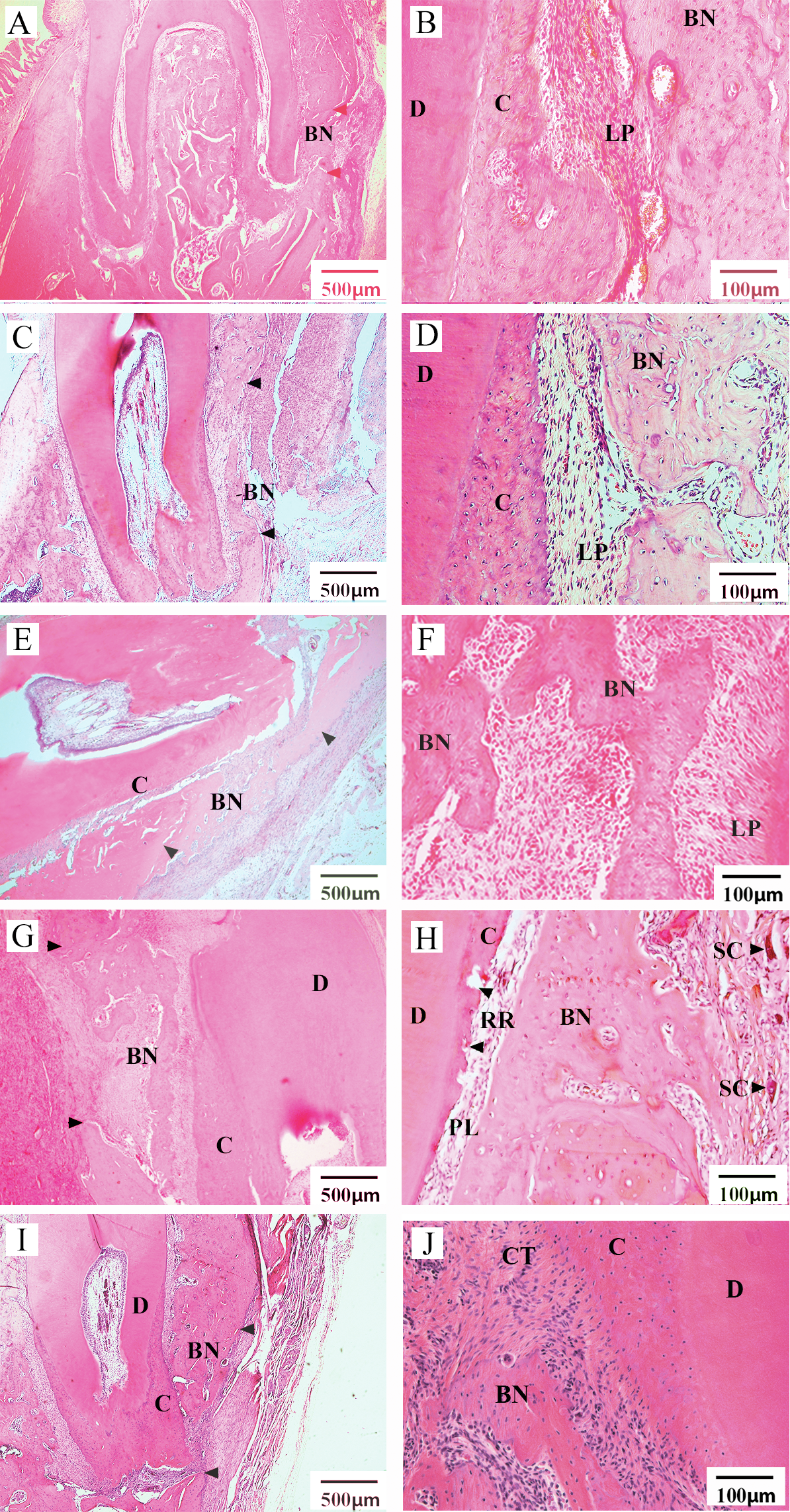

Supplement: S3 Fig — Images A and B indicate the use of PLLA/COL/HA membranes. Images C and D show PLLA/COL/HA associated with SHEDs. Images E and F show PisPLLA/COL/HA membranes. Images G and H show PisPLLA/COL/HA membranes associated with SHEDs. Images I and J show the negative control, without the use of the materials or cells. In images A, C, E, G and I the insets show the edges of the periodontal defect. New bone formation (BN), periodontal ligament (PL), cementum (C), dentin (D), conjunctive tissue (CT), scaffolds (SC), and root resorption (RR) are indicated in the figures. (TIF) [file pone.0152412.s003.tif]
